# Thermal stability analyses of human PERIOD-2 C-terminal domain using dynamic light scattering and circular dichroism

**DOI:** 10.1371/journal.pone.0221180

**Published:** 2020-04-22

**Authors:** Yuejiao Xian, Brenda Moreno, Victoria Miranda, Neha Vijay, Luis C. Nunez, Jennie Choi, Christian S. Quinones, Paulina Rios, Neha Chauhan, Karla V. Moriel, Noah J. Ruelas, Adan E. Castaneda, Ruben Cano Rodriguez, Bianca N. Amezaga, Seham Z. Azzam, Chuan Xiao

**Affiliations:** Department of Chemistry and Biochemistry, The University of Texas at El Paso, El Paso, Texas, United States of America; Russian Academy of Medical Sciences, RUSSIAN FEDERATION

## Abstract

At the molecular level, the circadian clock is regulated by a time delayed transcriptional-translational feedback loop in which the core proteins interact with each other rhythmically to drive daily biological rhythms. The C-terminal domain of a key clock protein PER2 (PER2c) plays a critically important role in the loop, not only for its interaction with the binding partner CRY proteins but also for the CRY/PER complex’s translocation from the cytosol to the nucleus. Previous circular dichroism (CD) spectroscopic studies have shown that mouse PER2c (mPER2c) is less structured in solution by itself but folded into stable secondary structures upon interaction with mouse CRYs. To understand the stability and folding of human PER2c (hPER2c), we expressed and purified hPER2c. Three oligomerization forms of recombinant hPER2c were identified and thoroughly characterized through a combination of biochemical and biophysical techniques. Different to mPER2c, both thermal unfolding DLS and CD analyses suggested that all forms of hPER2c have very stable secondary structures in solution by themselves with melting temperatures higher than the physiological body temperature, indicating that hPER2c does not require CRY to fold. Furthermore, we examined the effects of EDTA, salt concentration, and a reducing agent on hPER2c folding and oligomerization. The ability of hPER2c forming oligomers reflects the potential role of hPER2c in the assembly of circadian rhythm core protein complexes.

## Introduction

In mammals, the circadian rhythm is driven by an intrinsic 24-hour biological clock that synchronizes the sleep-wake cycle to external environmental cues such as light [1–4]. Over the past three decades, core proteins that regulate the circadian rhythm have been identified including Brain and Muscle ARNT Like-1 (BMAL1), Circadian Locomotor Output Cycles Kaput (CLOCK), Cryptochrome (CRY), and Period (PER) [4]. Being transcription factors, CLOCK and BMAL1 heterodimerize and activate many clock and clock-controlled genes that possess E-box elements in their promoter regions, including *Per* and *Cry* genes. After transcription and translation with time delays, PER and CRY proteins accumulate in the cytosol and form complexes that translocate into the nucleus to inhibit the function of the CLOCK/BMAL1 complex. Thus, PERs and CRYs regulate their own transcriptions to generate a negative feedback loop [4, 5]. Long term disruption of the circadian rhythm in humans have been associated with sleep disorders, heart diseases, hypertension, diabetes, higher risks for cancers, and other metabolic disorders [3, 6–11].

**Fig 1:**
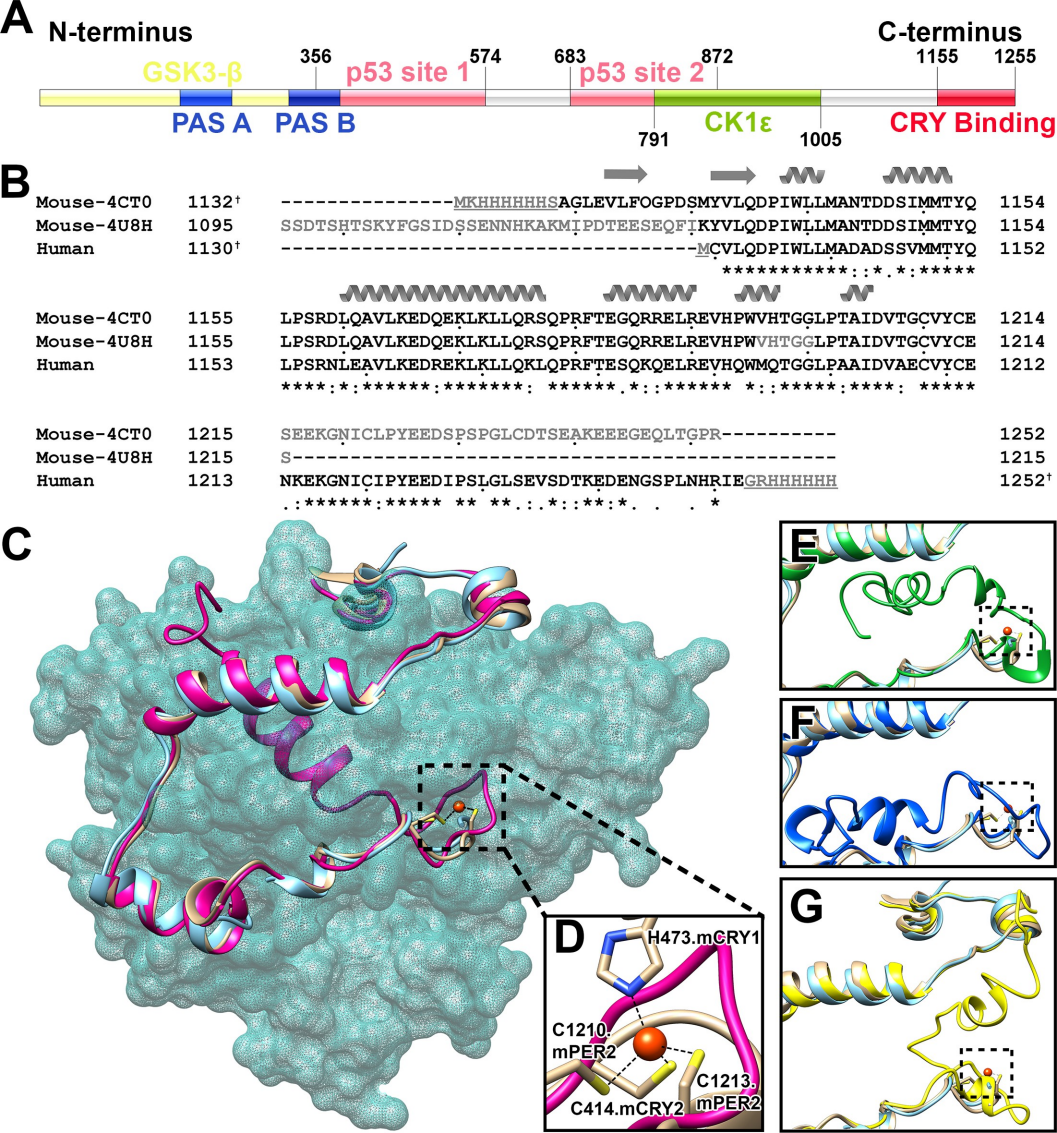
The structures and sequences of PER2. (A) Schematic diagram of human PER2 domain structure. The two PAS domains were colored in blue. The N-terminal possible phosphorylation region of hPER2 by GSK3-β was colored in light yellow and overlapped both PAS domains until residue 371. The two potential interaction regions of hPER2 with p53 were colored in pink with the residue range labeled on the top of both ends. The second interaction site is overlapped with casein kinase 1ε (CK1ε) phosphorylation regions that is colored in green and boundary residues were labeled on the bottom of both ends. The hPER2c region that interacts with CRY was colored in red with boundary residues labeled on the top. The diagram was generated by IBS [27]. (B) Multiple sequence alignments of mouse and human PER2c. Cloned sequences of mouse PER2c in PDB 4CT0 and 4U8H were aligned with that of human PER2c. Sequences whose structures are not available (not shown) in (C) are in light grey. Sequences that were added for cloning purpose are underlined. Sequence numbers marked with “†” do not include those residues added for cloning purposes. Dots are added below each 10^th^ residue for easy counting. α helices (helical ribbon) and β strands (arrows) are labeled above the corresponding sequences. “*”, “:”, “.”, and space beneath the aligned sequence indicate identical (conserved), strongly similar, weakly similar, and different (not conserved), respectively [28]. (C) Comparison between the selected Model 3 from I-TASSER homology modeling server of hPER2c and two structures of mPER2c solved in the complexes with mouse CRYs. Two mPER2c structures have been solved in complex with mCRY1 (PDB ID 4CT0, colored in orange) and mCRY2 (PDB ID 4U8H, colored in cyan). Using Chimera [29], these two mPER2c structures have been superposed on the homology model of hPER2c (colored in pink) with extra C-terminal parts that have no available crystal structures. The structure of mCRY2 (from PDB 4U8H) is presented with a transparent light sea green surface to show how these PER2cs are wrapped around CRY. The interface region between mCRY1 and mPER2c where the Zn^2+^ (red sphere) is located (chelated by four residues from both molecules) was outlined in black dashed lines and enlarged in (D). The small panels of (E), (F), and (G) are similar but enlarged diagrams as (C) showing the C-termini (each of 40 residues) of Model 1 (green), 2 (blue), and 4 (yellow) of hPER2c from I-TASSER server, respectively. mCRY2 density was removed for clarity. The locations of Zn^2+^ (red sphere) in these models were outlined in black dashed lines.

In humans, PER protein has multiple paralogs known as hPER1, hPER2, and hPER3 [12, 13], while CRY protein has two paralogs as hCRY1 and hCRY2 [14, 15]. Among these three PERs, hPER2 has been implicated in familial advanced sleep phase syndrome due to a single point mutation (S663G) [16]. hPER2 has multiple functional domains that are known to interact with diverse proteins and activate many pathways related to various disorders and diseases [10, 11]. For instance, hPER2 has been shown to interact with p53, the tumor suppressor protein, at two different regions [17–19] (Fig 1A). The N-terminal region of PER2 is composed of two PAS (PER-ARNT-SIM) domains that overlap with GSK3-β phosphorylation sites, while the middle region of PER2 has casein kinase 1 δ or ε (Fig 1A) phosphorylation sites[16, 20–22]. In rats, the C-terminal region of PER2 (PER2c) has been previously shown to play a key role with CRY1 in translocating the PER2/CRY1 complexes into the nucleus [23]. PER2c is also predicted to act as a Nuclear Localization Domain (NLD) [24]. In this study, we focus on the characterization of hPER2c molecule *in vitro* using size exclusion chromatography (SEC), transmission electron microscopy (TEM), dynamic light scattering (DLS) [25], and circular dichroism (CD) spectroscopy [26].

Based on previous mutagenesis studies, amino acid residues between 1179 and 1198 in rat PER2c are believed to be essential for binding to CRY1. Substitutions or deletions of the residues in this conserved region have shown to affect PER2’s functions, which was predicted to be due to the changes in its structure [23]. Following the mutagenesis studies in rats, two structures have been solved for mouse PER2c (mPER2c) in their complex forms, one with mouse CRY1 (mCRY1) [30] and the other with mouse CRY2 (mCRY2) [31]. In the structural study of mCRY1 (residue 1 to 496) and mPER2c (residue 1132 to 1252) complex, the corresponding electron densities for residues from 1215 to 1252 in mPER2c were too weak to determine their structures (Fig 1B and 1C). In another structural study, mCRY2 (residue 1 to 512) was complexed with mPER2c (residue 1095 to 1215). The recombinant mPER2c had 26 residues longer sequence at the N-terminus (1095–1131) and 37 residues shorter at the C-terminus (1216–1252) when compared to the mCRY1/mPER2c crystal structure (Fig 1B). Although in the study of mCRY2/mPER2c, the recombinant mPER2c has extra residues (1095–1131) at its N-terminus, there is no structure available for these residues. It is noteworthy that in both studies, mPER2c has a highly extended structure that embraces mouse CRYs with five to six α-helices (Fig 1C). The two complex structures are very similar to each other even though one is mCRY1 and the other is mCRY2. The RMSDs between the two mCRYs are 1.84Å over all the 482 matching pairs of Cα atoms and 1.59Å over the 78 match pairs of Cα atoms between the two mPER2c in the complexes (Fig 1C).

The crystal structures have provided rich information about the static structures of CRY and PER as well as their interactions, however, they cannot elucidate the dynamics of the proteins in solution. The structural study of mCRY1/mPER2c included CD analyses of each individual component as well as the complex [30]. The CD data of unbound mPER2c showed much less secondary structure compared to that in the mCRY1/mPER2c complex. Therefore, the authors had speculated that mPER2c becomes significantly structured after interacting with mCRY1 [30]. It is noteworthy that these CD data were measured only at 4°C without characterizing the structures at other temperatures.

Sequence alignment data showed that mouse and human PER2c are highly conserved with approximately 69% identical residues and 83% similarity between residues 1130 to 1250 of human sequence (Fig 1B). Here, we expressed and purified the human PER2c (hPER2c). Three different oligomerization forms of hPER2c were identified by both SEC and TEM. We then measured the CD and DLS spectra of all three forms of recombinant hPER2c at various temperatures to characterize their structural dynamics. Based on our results, all three forms of hPER2c in solution by themselves maintain highly ordered secondary structures, suggesting that hPER2c does not require CRY to facilitate the folding. Furthermore, our study suggested a strong relationship between all three different oligomers, providing insights for hPER2c’s roles in the formation of circadian complexes.

## Materials and methods

### Homology modeling of hPER2C

Sequence alignments of mPER2c to hPER2c were performed with CLUSTAL Omega [28] to determine their domain similarity. Since the crystal structure of hPER2c is not solved, we modeled the hPER2c 1130–1252 residues using I-TASSER server [32], which provided multiple models that were close to the available mPER2c structures. The I-TASSER modeled hPER2c structure was superposed to mPER2c structures using Chimera [29], which also provided RMSD results. PROCHECK[33] was used to validate the models.

### Gene cloning

Based on the structural study of mPER2c/mCRY1 [30], the gene sequence corresponding to residue 1130 to 1252 of hPER2c was amplified by PCR using a Phusion Flash High-Fidelity PCR Master Mix (Thermo Fisher Cat. #F548S). The PCR amplified fragment was gel purified using the QIAquick Gel Extraction Kit (Qiagen Cat. #28704) and ligated into the expression vector pCold I (Takara Cat. #3361) with NdeI and XhoI (NEB Cat. #R0111S and R0146S) overnight at 16°C using T4 DNA ligase (NEB Cat. #M0202S) before transformation into *E*. *coli* BL21(DE3)pLysS competent cells.

### Protein expression

Individual colonies were picked and grown into a starter culture in 3mL LB media with ampicillin (100μg/ml) overnight at 37°C. A large-scale culture was inoculated from the overnight pre-culture at a 1:1000 dilution. The culture was grown at 37°C until an OD_600_ of 0.6 was reached. The temperature was then reduced to 16°C to activate the cold shock promoter in the vector. At OD_600_ 0.7, the culture was induced with 2mM IPTG (Isopropyl β-D-1-thiogalactopyranoside). After four hours, the cells were harvested by centrifuging 4,690xg at 4°C for 30 minutes and the pellets were resuspended in lysis buffer (20mM Tris-base pH 7.5, 500mM NaCl, 0.3mg/mL lysozyme, 1mM EDTA) and sonicated to release protein from bacteria. The lysate was centrifuged at 44,000xg for 45 minutes to remove the cell debris. The supernatant was filtered using a 0.22μm filter prior to loading onto the affinity column.

### Affinity chromatography

Affinity chromatography purification was performed using an AKTA PURE chromatography system (GE Healthcare Life Sciences) and a 5ml HisTrap HP column (GE Healthcare Life Sciences Cat. #17-5248-02). Binding buffer (20mM Tris-base pH 7.5 and 500mM NaCl) was used to equilibrate the column prior to loading the samples. After loading, the column was washed twice with 50mM imidazole and 100mM imidazole under the same pH and salt conditions as the binding buffer. Finally, the bound hPER2c was eluted with elution buffer (20mM Tris-base pH7.5, 500mM NaCl, and 300mM imidazole). The elution peak was collected and run on a 10% SDS-PAGE for analyses. Bands on SDS-PAGE were cut and collected for mass-spectrometry analyses at the Taplin Mass Spectrometry Facility at Harvard Medical School using LC/MS/MS to verify the recombinant protein sequence.

### Size exclusion chromatography

The purity and size of recombinant hPER2c were verified using SEC. The AKTA PURE chromatography system is kept in the 4°C within a refrigerated cabinet. Prior to loading the samples, the SEC (HiLoad 26/600 Superdex 200 pg, GE Healthcare, Cat. #28989336) was equilibrated with buffer (20mM Tris-base, pH7.5, 50mM NaCl) and calibrated with selected standards from the SEC Markers Kit (Sigma Cat. # MWGF200-1KT) and a High Molecular Weight (HMW) SEC Calibration Kit (GE Healthcare, Cat. # 28403841). The collected hPER2c samples from affinity chromatography were then loaded to the SEC column. The eluted hPER2c fractions were then collected for SDS-PAGE analysis. The pure hPER2c fractions for each peak were then concentrated for DLS, CD and TEM analysis using Amicon concentrator with 3kD cutoff (Millipore Cat. #UFC900324). In order to understand the role of reducing agents in hPER2c’s stability and oligomerization, 20mM DTT was added to the concentrated hPER2c fractions. Following the DTT treatment for 36hrs, the hPER2c samples were loaded to analytical SEC (Superdex 200 Increase 10/300 GL, GE Healthcare, Cat. #28990944), which was calibrated with selected standards from SEC Markers Kits as mentioned above and equilibrated with buffer containing DTT (20mM Tris-base, pH7.5, 50mM NaCl, 20mM DTT). Samples without any DTT treatment served as a control and were analyzed with the same SEC with DTT in the buffer.

### Transmission electron microscope

Elution fractions of the recombinant hPER2c were diluted to 0.05mg/ml using the same buffer as in SEC prior to negative staining. For each diluted elution, 4μl of sample was added onto an ultrathin carbon film on Lacey 400 mesh grid (Ted Pella Inc., cat #1824), which was glow discharged using Emitech K950 carbon coater with K350 glow discharge unit for 1.5min with a current of 15mA. The sample was incubated on the grid for 3min while the grid rested about 2.5cm above ice. The excess sample was then blotted using filter paper and stained with 4% Thulium Acetate (Sigma-Aldrich, cat # 367702) for 1.5 mins. The stained sample was then imaged at 300 kV with a 120,000x nominal magnification (calibrated as 137,800x) on a JEOL JEM-3200FS transmission electron microscope equipped with a field emission gun and an in-column omega filter. The micrographs were recorded on a Gatan 4k x 4k UltraScan US4000 CCD camera with defocus values at approximately 2μm. Such TEM analysis were also carried out on the sample treated with DTT for 36hrs for the purpose of visualizing the effects of a reducing environment on the oligomerization of hPER2c.

### Dynamic Light Scattering (DLS)

To assess the hydrodynamic diameters of the purified hPER2c from SEC, the corresponding DLS spectra were measured via a Zetasizer Nano S (Malvern) using a cuvette with a 1cm path length at various temperatures ranging from 4°C to 85°C (Temperatures above 85°C would lead to protein aggregation and good spectra couldn’t be obtained). Refractive index of the cuvette material was 1.59 while that of the dispersant was 1.33. The viscosity of the dispersant was 1.57. For each measurement, the sample was incubated at the desired temperature for 3mins. The DLS data were averaged from 11 iterations. All hPER2c samples were concentrated to approximately 0.7mg/ml for thermal stability studies. To investigate the effect of salt on different forms of hPER2c, the salt concentration was gradually increased while the size of the protein was monitored by DLS. To examine the effect of Zn^2+^ on hPER2c folding, 10mM EDTA was added to all three forms of hPER2c and incubated at room temperature for 12hrs and 36hrs, then their size was measured by DLS and compared to the control without adding any EDTA. In addition to SEC and TEM analyses mentioned above, the effects of reducing agent on the hPER2c were also investigated by DLS at 12hrs and 36hrs while compared to the control without any DTT.

### Circular dichroism

Far-UV CD measurement of the recombinant hPER2c was conducted using a J-1500 CD spectrophotometer (JASCO) connected to a Peltier temperature controller. Elution fractions of recombinant hPER2c from SEC were concentrated to approximately 15μM in the same SEC buffer (20mM Tris-base buffer, pH 7.5, 50mM NaCl). For each measurement, 400μl of sample was added in a 0.1cm path length rectangular quartz cuvette (JASCO, cat#0556). The CD ellipticities (θ_obs_) were then measured from 190nm to 260nm with the scanning speed set as 200nm/min and data integration time (D.I.T) set as 1sec. Buffer samples without protein were used as base-line measurements. The final ellipticity spectrum was averaged from 5 baseline-corrected measurements to improve signal to noise ratio. The ellipticities (θ_obs_) measured by the CD spectrophotometer were used to calculate mean residue ellipticity in the formula [θ] = θ_obs_/*cnl* where, *c* stands for the concentration of protein in moles, *n* for the number of residues and *l* for the path length of the cuvette. Furthermore, thermal denaturation studies were carried out by heating each sample from 5°C to 90°C with 5°C intervals, and the ellipticities were measured using the same parameter settings described above. At each temperature, the sample was equilibrated for 3 minutes prior to the CD measurements. The thermal unfolding profile of each sample was characterized using the mean residue ellipticity minima at 208nm ([θ]_208_) to determine *T*_*m*_ values by fitting the Boltzmann sigmoid equation to [θ]_208_ using Origin (Version 8, OriginLab Corporation). The spectrum was further analyzed using BeStSel software to determine secondary structure content [34, 35]. In addition to BeStSel, DichroWeb, K2D3 web server and the JWMVS-529 CD Multivariate SSE program (JASCO) were also used to validate the secondary structure content. The CD spectra for different oligomerization forms of hPER2c were deposited into the Protein Circular Dichroism Data Bank at (http://pcddb.cryst.bbk.ac.uk/) having accession ID from CD0006240000 to CD0006242000.

## Results

Consistent with our sequence alignment, the template with the highest similarity to hPER2c estimated by I-TASSER is the mPER2c structure complexed with mCRY1 (PDB ID 4ct0) with sequence coverage of 68%. I-TASSER server provided the five best homology models of hPER2c with C-scores (confidence score) [36] of -2.33, -2.74, -2.36, -2.86 and -3.00, respectively (S1 Table). The C-score should between -5.0 to 2 with the higher the value, the higher confidence of the model [36]. Since hPER2c shares very high homology (approximately 69% amino acid sequence identity) to mPER2c (Fig 1B), it is not surprising that the N-terminal 83 residues (residue 1130 to 1212) of the top four homology models of hPER2c superposed with the solved mPER2c structures very well (Fig 1C and 1E–1G) with Cα RMSD all less than 1.0Å over 83 atom pairs to mPER2c atomic structure (PDB 4CT0) (S1 Table). Model 5 from I-TASSER server has the worst C-score and was unable to be superposed to the mPER2c well (the best RMSD of superposing was more than 15Å), thus, we will not discuss it further. The I-TASSER server also provided the models of hPER2c for the C-terminal extra residues from 1213 to 1252 with some putative helical regions (Fig 1C and 1E–1G). The major differences among the best models from I-TASSER server exist in this region. However, since this part does not have X-ray structures to serve as templates, the modeled structures of these C-terminal 40 residues are purely speculative. Since the goal of our homology modeling is to predict the structure of hPER2c in solution by itself in absence of hCRYs, multiple models of this extra part clashed with mCRY1 in the complex structure (Fig 1C and 1E–1G), indicating that in order to interact with CRYs, this part needs to be relocated. Among the top four models, Model 3 possesses the second highest C-score, second lowest RMSD value when superposed with mPER2c structure, and lowest total number of residues in disallowed and generously allowed regions of Ramachandran plot (S1 Table). Model 3 is currently the best prediction of the hPER2c structure in our homology modeling (Fig 1C). Regardless, we still present all the other best models to show multiple possible structures of the extra C-terminal part of the hPER2c (Fig 1E–1G).

**Fig 2:**
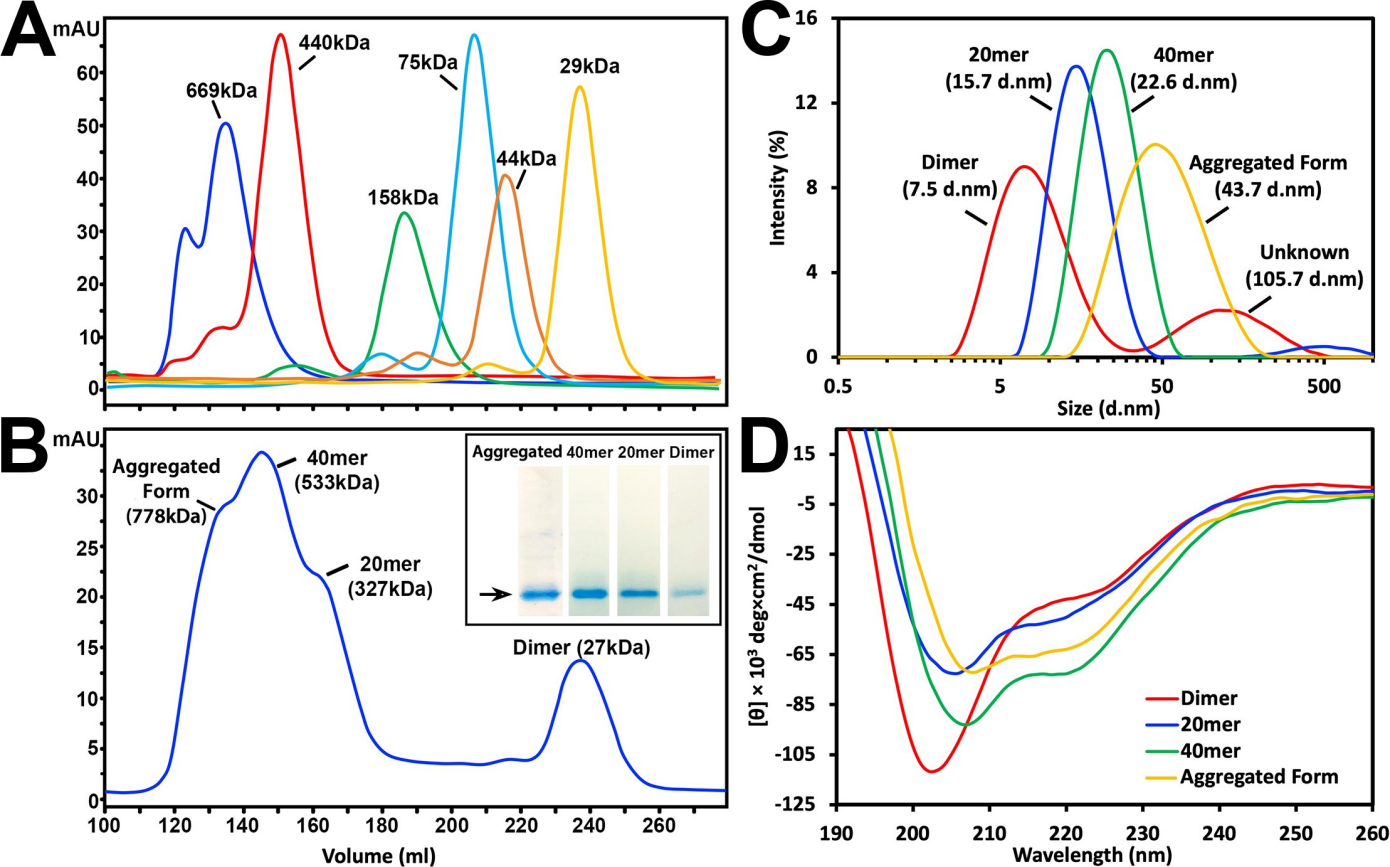
Purification of hPER2c by SEC and DLS/CD profile of all four forms of hPER2c. (A) Chromatogram of molecular standards: thyroglobulin 669kDa (blue), ferritin 440kDa (red), aldolase 158kDa (green), conalbumin 75kDa (cyan), ovalbumin 44kDa (orange), and carbonic anhydrase 29kDa (yellow). (B) Chromatogram of hPER2c purification showing four different peaks at 778kDa, 533kDa, 327kDa, and 27kDa. Based on calibration in (A), these four peaks were named as aggregated form, 40mer, 20mer, and dimer as described in main text and used in the following panels. Coomassie blue stained 10% SDS-PAGE gel of purified recombinant hPER2c for each form were show on the side. Most of them except the aggregated form showing a single band. (C) DLS spectra of peak fraction from SEC in (B). The peaks for aggregated form, 40mer, 20mer, and dimer are labeled with their corresponding measured hydrodynamic radius. (D) CD spectra of peak fraction from SEC in (B). In both panel (C) and (D), the DLS and CD Spectra of aggregated, 40mer, 20mer, and dimer forms of recombinant hPER2c were colored in yellow, green, blue, and red, respectively.

Recombinant hPER2c was purified to high purity by affinity chromatography and SEC (Fig 2B) to exclude the influence of major contaminants to the DLS and CD data. Four different forms of recombinant hPER2c were showed during SEC purification, whose hydrodynamic radii were then measured by DLS. Based on the molecular weight estimation by calibration (Fig 2A), these four forms (Fig 2B) are named as aggregate form, 40mer, 20mer, and dimer, respectively. The exact oligomeric numbers for the 40mer and 20mer were roughly estimated and might not be precise. The DLS spectrum of hPER2c for all four forms are shown in Fig 2C and their measured sizes are presented in Table 1. The polydispersity index is widely used in analyzing DLS data[25]. It is rarely smaller than 0.05 but any value above 0.7 indicates a broad size distribution and is not suitable for DLS analyses[25]. A PDI of 0.3 or less indicates a homogenous population of the sample whereas a PDI value range from 0.3 to 0.7 would suggest a polydisperse distribution of the sample[37]. In our measurements, this index for 40mer and 20mer are ranged between 0.166 and 0.265 representing a monodisperse sample [25] (Table 1). The PDI index of the hPER2c dimer is 0.401, and two size distributions (at 7.5nm and 105.7nm) are clearly visualized in its spectra, suggesting that the hPER2c dimer sample is polydisperse (Fig 2B) even eluted in a single peak from SEC purification. The longest distance between two atoms in mPER2c measured based on the mPER2c/mCRY1 complex (PDB 4CT0) is approximately 5.7nm, hence, the signal that appeared at approximately 7.5nm is likely to be the dimerized hPER2c. The DLS measurement was performed as soon as the sample was eluted from SEC, which should have separated any bigger complexes from hPER2c dimer. However, the 105.7nm signal persisted in all our duplicates as well as when the dimer was concentrated. It is noteworthy that the radio between the size of 105.7nm peak and that of the 7.5nm peak remained similar in all the samples (S1 Fig). Similar additional peaks have not been observed in other forms of hPER2c (Fig 2C and S1 Fig). The Y-intercept value is another commonly used parameter to assess the signal-to-noise ratio of DLS. A Y-intercept value above 0.6 is acceptable and a value above 0.9 indicates the best quality of data acquisition as suggested by the instrument manufactory white paper (Malvern Instruments Worldwide). Y-intercept values at all forms were higher than 0.6 again indicating good quality of the DLS data.

**Table 1:** The DLS measurement profiles of all four forms of hPER2c resulted from SEC purification.

	dimer	20mer	40mer	Aggregated form
**Z-average(d.nm)**	8.927	15.660	22.617	43.670
**Peak location (d.nm)**	7.531^a^	15.690	24.360	43.820
	105.700^b^			
**PDI**	0.401	0.265	0.166	0.206
**Intercept**	0.630	0.799	0.901	0.914

^a^ Location of the peak corresponding to the putative dimer

^b^ Location of the peak corresponding to the unknow complex persisting in the dimer sample

**Fig 3:**
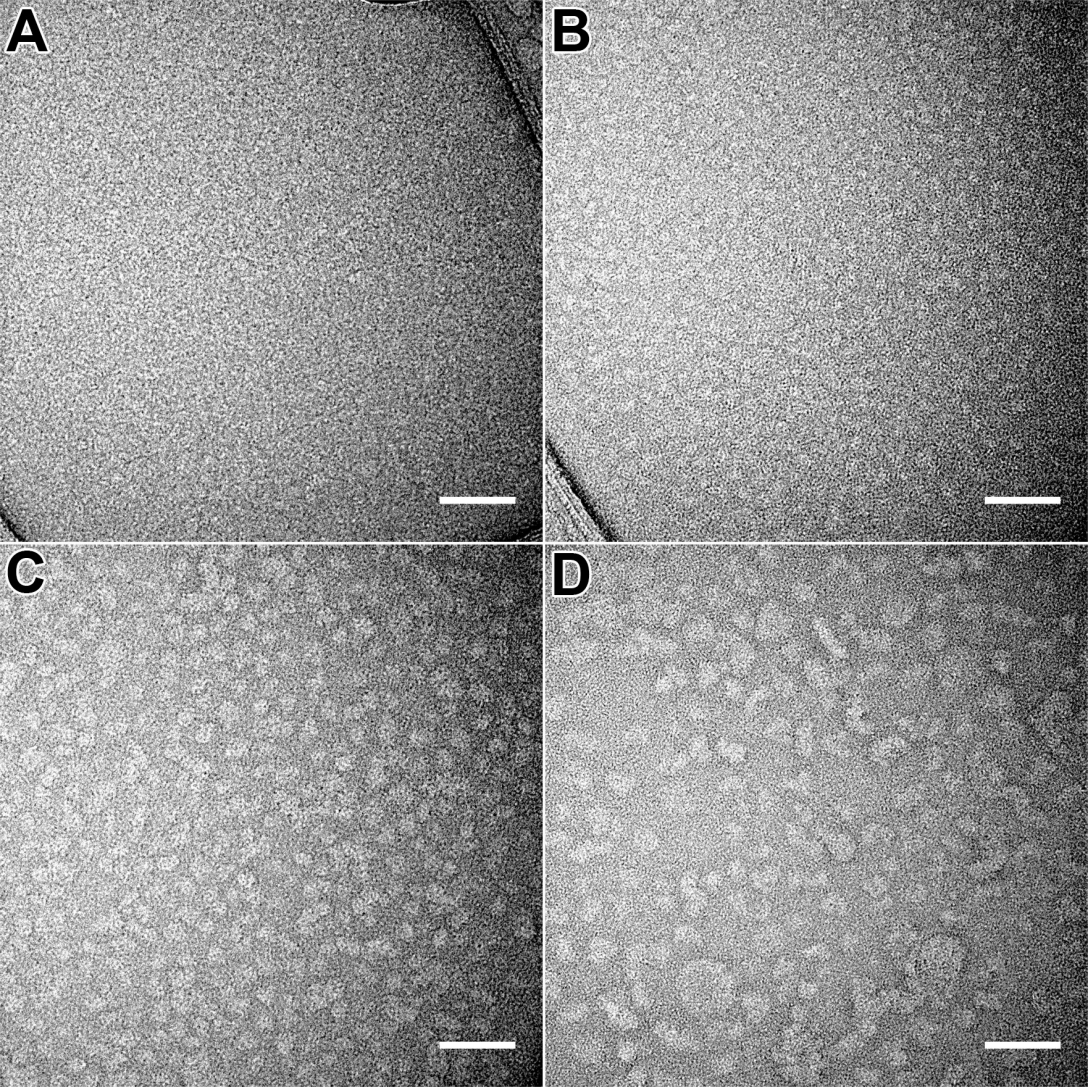
Negatively stained TEM images of all four forms of hPER2c. **(A)** dimer; **(B)** 20mer; **(C)** 40mer; and **(D)** aggregation. Scale bar is 50nm in all four panels.

The size of all four forms of hPER2c was further confirmed by TEM (Fig 3). The hPER2c 40mer and 20mer are homogeneous in size and have globular shape. Furthermore, the measured size of hPER2c aggregated form, 40mer, and 20mer showed consistency with the DLS measurements. Although based on the PDI and Y-intercept values, the aggregated form of hPER2c is also monodisperse, the width of the aggregated form on the logarithm presented DLS plot is much wider than those of the 40mer and 20mer. This indicates that the aggregated form of hPER2c has a much wider size distribution. It is consistent to TEM observations of the aggregated sample that has heterogeneous blobs with various size and shape. Therefore, the aggregated form would not be further discussed. The hPER2c dimer is hardly visible as the molecular weight of 29kDa is too small to be visible using current TEM techniques.

The CD spectra of all four hPER2c forms were analyzed by BeStSel in order to determine their secondary structure compositions (Table 2). The normalized root mean square deviation (NRMSD) values were widely used in the field to access CD data quality [24]. NRMSD measures how well the CD data (over the entire wavelength range of the measurement) match the theoretical CD spectra calculated from the derived secondary structure composition [24]. Ideally, NRMSD should be lower than 0.05, and practically, it should be less than 0.1. All the NRMSD values of the three forms of hPER2c in our measurements are lower than 0.05, indicating very good fits [24, 37].

**Table 2:** The secondary structure compositions of all three hPER2c forms estimated from their CD spectrum by BeStSel, in comparison to previous measurement and crystal structures.

PER2c type	Regular Helix(%) (distorted Helix)	Antiparallel Sheet(%) (distorted sheet)	Parallel Sheet(%)	Turns (%)	Unordered (%)	RMSD	NRMSD
**Dimer**	7.8(3.5)	0(30.9)	0	15	42.8	0.0933	0.01408
**20mer**	8.8(5.5)	3.3(21.5)	0	15.5	45.4	0.0286	0.01116
**40mer**	9.3(7.9)	0(15.3)	7.6	13.4	46.6	0.0258	0.00647
**mPER2c (1132–1252)**^**a**^** CD analyses at 4**°C	3(12)	10(11)	\	25	38	0.1	0.032
**mPER2C (1132–1252) in PDB 4TC0**^**b**^	32.1	6	\	6.7	55.2	\	\
**mPER2C (1095–1215) in PDB 4U8H**^**b**^	29.8	0	\	5.8	64.4	\	\
**hPER2C (1132–1252) in modeled structure**^**b**^	42.7	0	\	20.2	37.1	\	\

^a^ CD spectra interpretation done previously by Schmalen I. el al. while mPER2c is binding to mCRY1/2 [25].

^b^ Secondary structure determination from 3D coordinates by WHAT IF program[38] using the DSSP algorithm[39], assuming no additional secondary structures in the regions between residue 1095 and 1130 (4U8H) and between residues 1215 to 1252(4CT0).

Previous studies speculate that the mPER2c domain gains secondary structure upon interacting with mCRY1 [30]. The CD data of mPER2c at 4°C was interpreted to have 3% regular helices with 12% distorted helices, 10% regular sheets with 11% distorted sheets, 25% turns, and 38% unordered [30](Table 2). Our hPER2c CD spectra at 4°C showed more regular helices in all three forms of hPER2c (7.8% for dimer, 8.85% for 20mer and 9.3% for 40mer), whereas the distorted helices of hPER2c dimer, 20mer and 40mer are 3.5%, 5.5%, and 7.9%, respectfully. No or very few regular sheets (0% for dimer and 40mer, 3.3% for 20mer) were detected. However, 30.9%, 21.5%, and 15.3% of distorted sheets were found in hPER2c dimer, 20mer, and 40mer, respectively. It is noteworthy that the percentage of both regular and distorted helices increase as the degree of oligomorization increases. On the contratry, the distorted sheets descreases as the degree of oligomerization increases (Table 2). Secondary structure esitmation done by DichroWeb, K2D3, and Jasco mSSE programs also show a similar trend (S2 Table).

**Fig 4:**
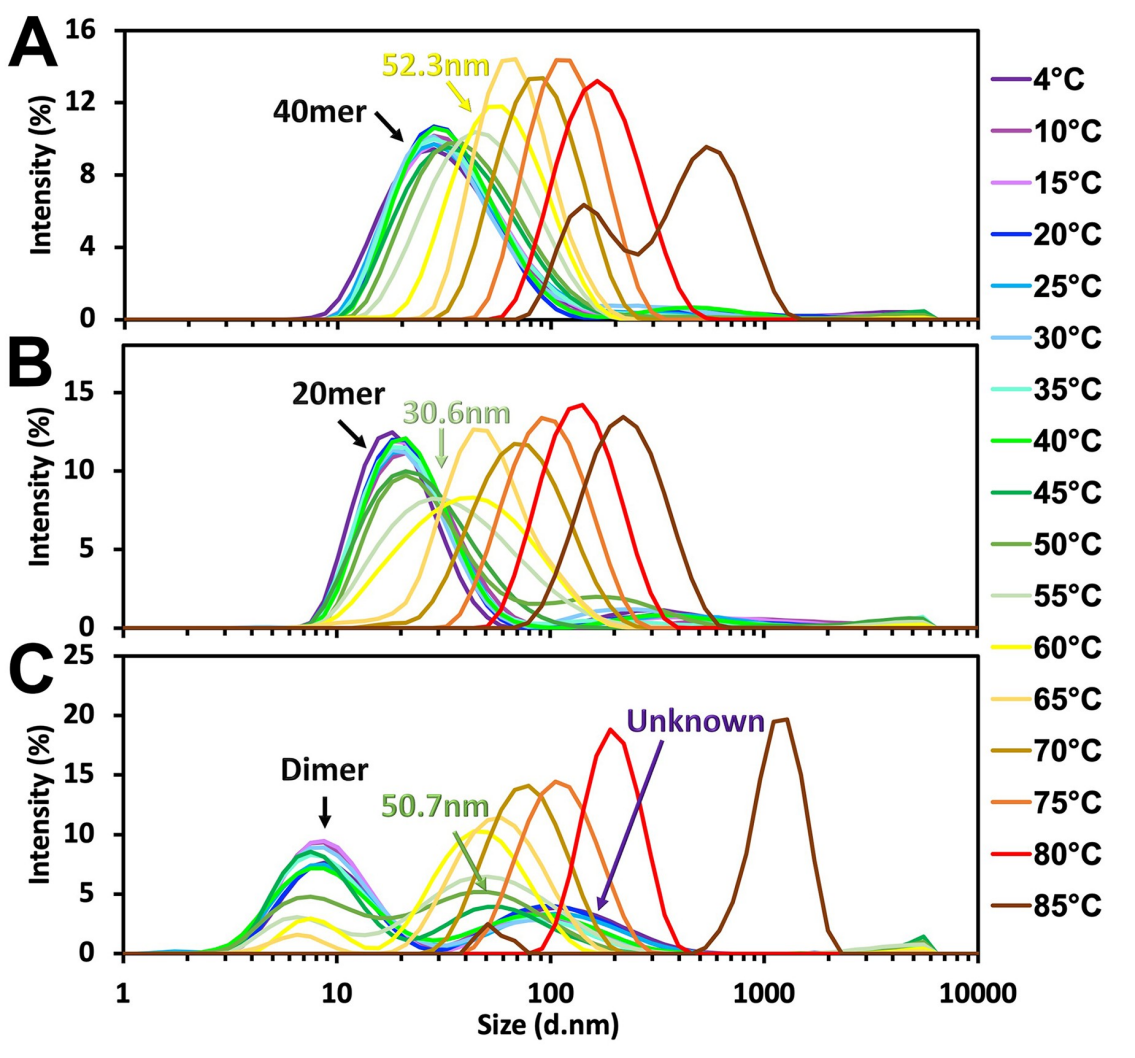
DLS thermal stability profiles of hPER2c 40mer **(A)**, 20mer **(B)**, and dimer **(C)** at gradually increased temperature in 5°C intervals. The DLS spectra were colored from purple to brown with increasing temperatures as indicated by the legend on the right. The peaks corresponding to the hPER2c 40mer, 20mer, dimer and the unknown complex at low temperatures are indicated with black arrows. The signal of significant size changes in all three samples were also indicated with their correspongding size.

The thermal stability of hPER2c dimer, 20mer, and 40mer were analyzed by DLS (Fig 4). The hydrodynamic diameters of all three forms of hPER2c corresponding to the increasing temperature are shown in S2 Fig and S3 Table. As the temperature increased, the hydrodynamic radiuses of all three forms of the hPER2c increased. Significant size changes were observed for hPER2c 40mer when the temperature reached 60°C, where its hydrodynamic radius increased from 42.1nm to 52.3nm (Fig 4A). As for hPER2c 20mer, the hydrodynamic radius increased (from 19.7nm at 4°C) to 30.6nm at 55°C (Fig 4B). The most dynamic changes were observed with hPER2c dimer, the 105.7nm peak at 4°C shifted to 50.7nm at 45°C. In the meantime, the intensity of the 7.5nm peak was reduced by approximately 44% at 50°C. At the same temperature, the intensity of the large diameter peak increased to the same level as the 7.5nm peak. At 70°C, the intensity of the 7.5nm peak deminished, while the remaining single peak continued to increase its diameter as the temperature raised (Fig 4C, S3 Table).

**Fig 5:**
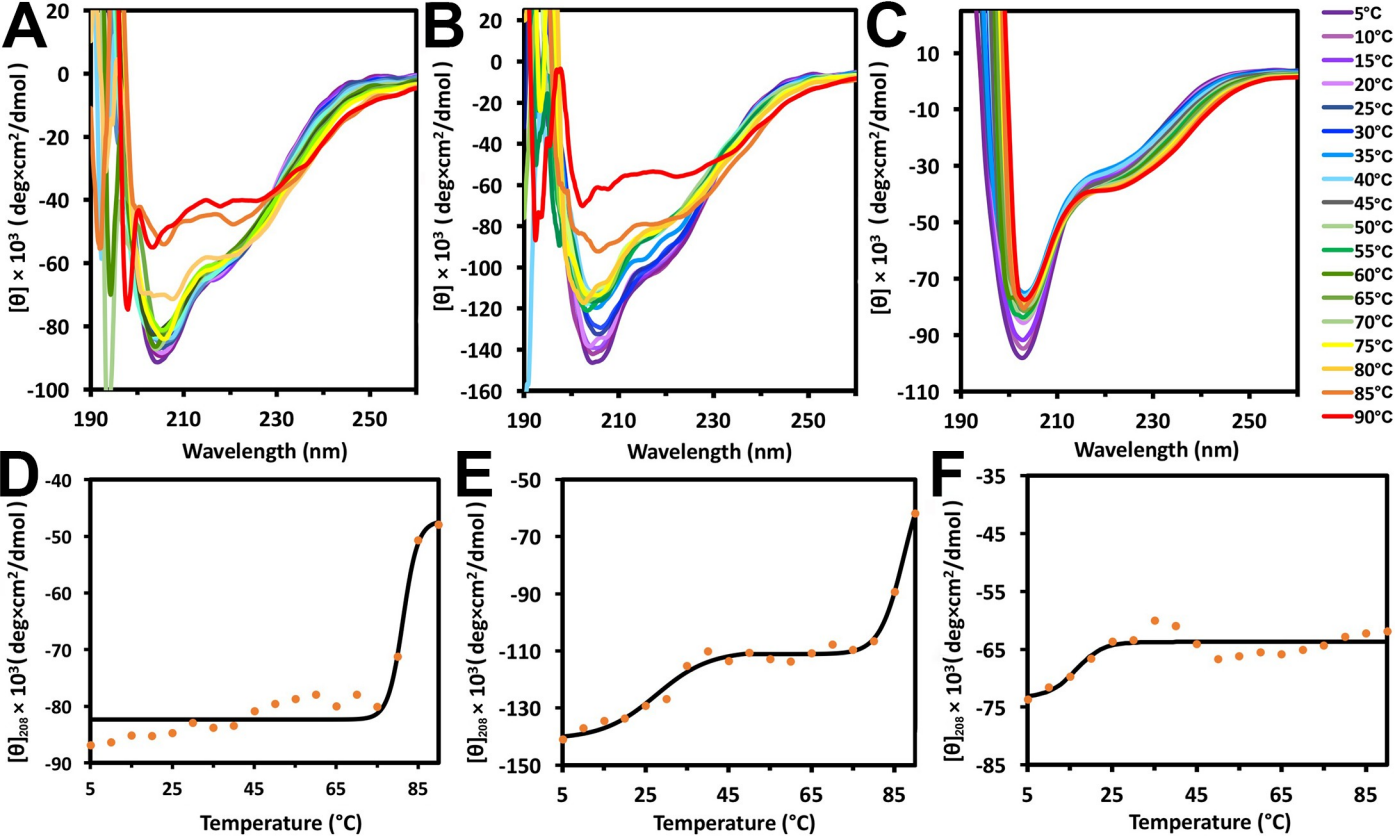
Far UV CD thermal unfolding profiles of hPER2c 40mer (**A** and **D**), 20mer (**B** and **E**), and dimer (**C** and **F**). Panel A, B, and C are the CD spectra at different temperatures ranging from 4°C (purple) to 90°C (red) for hPER2c 40mer, 20mer, and dimer, respectfully. The legend on the right shows the line colors and their corresponding temperatures. Panel D, E, and F are the values of mean residue ellipticity at 208nm ([θ]_208_) for hPER2c 40mer, 20mer, and dimer, respectfully, plotted against temperatures in orange dots. A curve (black solid line) has been fitted to [θ]_208_ to determine the T_m_ of the hPER2c 40mer, 20mer, and dimer, respectfully.

In addition, we also analyzed the CD spectra of all three forms of hPER2c at various temperatures ranging from 5°C to 90°C with 5°C intervals (Fig 5A, 5B and 5C). The ellipticities at 208nm ([θ]_208_) for all three forms of hPER2c were plotted against temperature and fitted with the Boltzmann sigmoid equation using Origin (Fig 5D, 5E and 5F) for determining their melting temperature T_m_. The T_m_ temperature of hPER2c 40mer was estimated to be 81.3°C with a standard error of 0.93°C, indicating the high stability of 40mer (Fig 5A and 5D). The [θ]_208_ of hPER2c 20mer clearly shows a transition state that appears at the temperature ranging from 35°C to 75°C. Fitting the ellipticity values of hPER2c 20mer from the native state to the transition state resulted the first T_m_ of 27.6°C (standard error 1.15°C), suggesting that the 20mer is likely to convert into the metastable transition state under physiological temperature. Fitting of ellipticity values of hPER2c from this transition state to a completely unfolded state resulted a second T_m_ of 87.3°C (standard error 2.20°C), indicating that the high stability of the transition state far beyond physiological temperature (Fig 5B and 5E). Suprisingly, there are minimal ellipiticity changes in hPER2c dimer even at high temperatures like 90°C (Fig 5C and 5F). CD measurement in water-based solution is limited to 90°C as the temperature is approching to the boiling point of water. As a result, the T_m_ temperature of hPER2c dimer couldn’t be accurately determined. This result indicated that the secondary structure of hPER2c dimer is highly stable with a T_m_ temperature beyond 90°C.

In order to understand the effects of salt concentration, reducing reagent, and divalent ions on the formation of hPER2c oligomerization, a series of studies were carried out using DLS, CD and TEM. The hydrodynamic radius of hPER2c 20mer and dimer were monitored as the NaCl concentration was gradually increased from 50mM to 500mM (S3 Fig), no significiant size changes were observed in both hPER2c 20mer and dimer.

**Fig 6:**
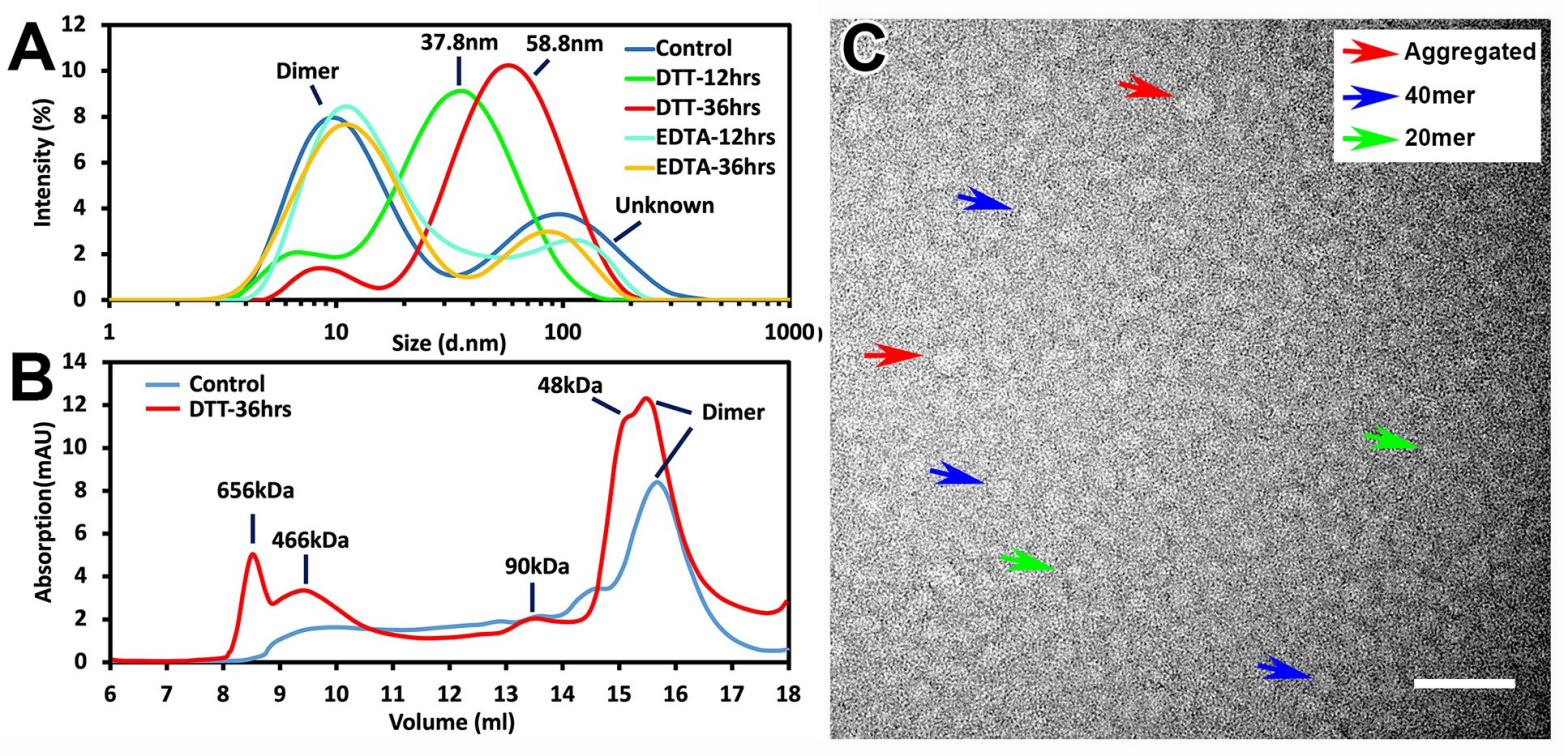
The effect of the DTT and EDTA on the oligomerization of hPER2c dimer. (A) The hydrodynamic size of hPER2c dimer monitored by DLS after incubation with DTT and EDTA for 12hrs and 36hrs. The peak for the dimer was labeled while the other additional peaks are labeled by their hydrodynamic diameters; (B) SEC chromatograms of DTT-treated hPER2c dimer (red line) and untreated one (blue line). The peaks of the dimer are labeled while the additional four peaks in the treated sample are labeled with their calibrated molecular weights; (C) The TEM image of DTT-treated hPER2c dimer. Molecules that resemble the hPER2c aggregation, 40mer, and 20mer were indicated using red, blue, and green arrows, respectfully.

As Zn^2+^ was suggested to play roles in mPER2c and mCRYs interaction[30, 31], it is of interest to investigate whether hPER2c acquires Zn^2+^ for its polymerization. Incubation of hPER2c 40mer, 20mer, and dimer in 10mM EDTA up to 36hrs, which should remove most bound Zn^2+^[40], did not lead to any significant size change when monitored by DLS (S4 Fig and Fig 6A).

To investigate if disulfide bonds play roles in hPER2c’s stability and oligomerization, all three forms of hPER2c were incubated in 20mM DTT for up to 36hrs, which was previously shown to be sufficient in reducing the disulfide bonds present in protein complexes[30]. Surprisingly, while the DTT treatment has no significant effects on hPER2c 40mer and 20mer, the hPER2c dimer was shown to have very interesting response to the presence of DTT. After incubating with 20mM DTT for 12 hours, the intensity of the 7.5nm peak significantly reduced and the 105.7nm peak deminished, while a strong signal appeared with hydrodynamic diameter the 37.8nm (Fig 6A). As the incubation time increased to 36hrs, the strong signal shifted to 58.8nm, close to the size of aggregation hPER2c. Note that the PDI for these messurement are all above 0.3, suggesting that the samples are still polydipersed, which indicates the presence of different forms of hPER2c. In order to get a more accurate measurement on the size distributions of the samples, the hPER2c dimer after DTT treatment for 36hrs were loaded to the analytical SEC (Fig 6B). Five elutions with distinct sizes were observed, whose calibrated sizes matches that of aggregated form (656kDa), 40mer (466kDa), hexamer (90kDa), tetramer/trimer (48kDa), and dimer, indicating hPER2c dimer becomes hetergenous in size after the DTT treatment (Fig 6B). The signal of the second elution corresponding to 40mer decayed slowly, indicating the presence of a smaller complex whose size might match with that of 20mer as shown in Fig 2B. The presence of various sizes of molecules was further verified through TEM imaging, where complexes that resemble the hPER2c aggregated form, 40mer, and 20mer were observed (Fig 6C).

## Discussion

Our homology modeling study shows it is likely that hPER2c shares similar structures to the N-terminal 83 amino acids as mPER2c. The structure prediction of the C-terminal 40 residues of hPER2c is difficult due to the lack of homology templates. However, multiple modellings of this region consistently suggest possible helical components (Fig 1C and 1E–1G, and S1 Table). In previous studies of mPER2c, the Zn^2+^ ion was found to play key roles at the interface between mCRY1 and mPER2c [30]. It is chelated by three Cysteine residues (two from mPER2c, one from mCRY1) and one Histidine residue (from mCRY1)[30] (Fig 1D). It is interesting to point out that this Zn^2+^ ion is located at the hinge region between the first 83 residues and the far C-terminal 40 residues (Fig 1C and 1E–1G), suggesting that it might have an influence on the conformation of the last part of hPER2c that needs to be relocated when interacting with hCRYs. Interestingly, our further investigation using EDTA shows that the Zn^2+^ is not required for hPER2c oligomerization (Fig 6A and S4B and S4D Fig).

Using multiple analytical techniques including SEC, DLS, and TEM, three different and stable forms of recombinant hPER2c were observed with molecular weights close to 40mer, 20mer, and dimer. These different forms of hPER2c were then further characterized in terms of thermal stability and secondary structure composition using DLS and CD. Thermal stability studies from both CD and DLS show that the hPER2c 40mer has a higher stability than 20mer. Our study here reveals that, under physiological temperature, hPER2c 20mer is likely to adapt to a metastable state (Fig 5E). Importantly, the secondary structure of hPER2c dimer are the most stable among all the three as no significant changes were observed at temperature as high as 90°C (Fig 5C and 5F).

Mouse PER2c was suggested to be largely unstructured and becomes highly ordered once it interacts with CRY1 [30]. In this previous study, the CD data indicated that mPER2c by itself was interpreted to be largely unstructured. However, our hPER2c CD spectra analyzed by multiple secondary structure estimation software consistently show that all three forms of hPER2c contain more regular helices and distorted sheets, but less distorted helices and no or very few regular sheets, when compared to mPER2c (Table 2 and S2 Table). As the degree of oligomerization increases, the percentage of both regular and distorted helices increase, whereas the distorted sheets decrease. These results suggesting that the oligomerization of hPER2c can result in more helical structures. The α-helical percentage in the crystal or homology modeled structures are 35% and the β-sheet percentages in those structures are close to zero (Table 2) indicating the CD estimation has large errors compared to the real structure. Our secondary structure prediction from the CD data is closer to those calculated from the crystal or modeled structures (Table 2) that do not contain β-sheet. Nevertheless, CD can measure the dynamic structural changes among different oligomers as well as their stability against temperature in real time with a small quantity of sample. This ability provides rich information about the secondary structural stability of hPER2c, which is different to that of mouse orthologs.

It has been speculated that mammalian PER2 could form a homodimer with the rich coil-coil structure near the C-terminal domain [41]. In our study, the dimerized hPER2c was not only successfully purified, but also characterized through a series of thermal stability analysis techniques. During our DLS studies, we observed a persistent peak at different conditions (salt and protein concentrations). This peak corresponds to a large complex with a hydrodynamic size of 105.7nm. However, this complex was not detected by SEC. We speculate that this complex could be a loosely associated form of hPER2c dimers, which dissociates during the SEC but quickly re-associate after eluted from SEC and then can be detected by DLS. This speculation is supported by the ratio between the two DLS peaks maintaining the same as the protein being concentrated (S1 Fig) indicating an equilibrium between these two forms of the hPER2c dimer. This is further supported by the absence of any large structures on the TEM images of dimer complexes (Fig 3A). However, we did not rule out the possibility that the sample preparation for TEM would remove such large complexes. As described in the result section, based on the DLS thermal dynamic measurements, the equilibrium between the hPER2c dimer and the weakly associated form changed. It first merged into a single peak with a size of 50.7nm and maintained the size until 70°C, then slowly increased the size when the temperature increased (Fig 4C).

To investigate whether disulfide bonds involve in stabilities of all three forms of hPER2c, they were treated with 20mM DTT for up to 36hrs. No effects on 40mer and 20mer were observed. However, both DLS, SEC and TEM showed the appearance of oligomers and aggregated forms after DTT treatment of the hPER2c dimer (Fig 6). The level of aggregation increased as the DTT incubation time increased (Fig 6A). We conclude that the disulfide bonds are involved in stabilizing the hPER2c dimer and prevent it to form stable oligomers. Disruption of these disulfide bonds allows further oligomerization of hPER2c. The expression of recombinant hPER2c occurred inside the cytoplasm of *E*.*coli*, where a reducing environment prevents the formation of the disulfide bonds. We speculated that the dimeric hPER2c could be an artificial product introduced during harvesting/purification process *in vitro*, where the reducing potential wasn’t sufficient to prevent the formation of the disulfide bonds. The dimer of hPER2c with disulfide bonds should not exist in an eukaryotic cytosol which also has a reducing environment. Based on these observations, without the disulfide bonds, the hPER2c will form large oligomers. This is consistent to the observation that the hPER2c 40mer and 20mer are produced in the high concentration in our expression system.

As mentioned above, both CD and DLS thermal stability studies show that both 40mer and 20mer have high stability with melting temperatures. Furthermore, our secondary structural analyses show that both hPER2c 40mer and 20mer contained more helices and less strands when compared to the dimeric hPER2c. Overall, hPER2c 40mer has higher percentage of helices and better stability than 20mer. We speculate these two oligomer forms were stably formed with helical coil-coiled structures. Wheel plot analysis of the longest helix in hPER2c modeled structure (residue 1158 to 1175) showed a unique amphipathic property (S5 Fig) with one side very hydrophobic and the other side with alternating positively and negatively charged residues. This characteristic property can facilitate a large bundling of helices via hydrophobic and charge-charge interactions. Furthermore, these oligomer forms were not affected by DTT treatments meaning that disulfide bonds do not play roles in their formations. Instead, the disulfide bond formation might keep hPER2c in dimeric form as discussed above.

It is noteworthy that the oligomerization of hPER2c only results in two different oligomeric forms with distinct molecular weights close to 40mer and 20mer instead of a continuous size distribution. In a recent study on the assembly products of the core circadian clock proteins in mouse liver cell[42], large circadian rhythm core protein complexes were identified. The study also emphasized the central role of hPER2 in forming these circadian protein complexes. Based on our characterization, hPER2c tends to form large oligomers in reducing environments such as inside cytosol. Therefore, the hPER2c part can be the core for forming large circadian complexes. While the mechanism of hPER2c oligomerization resulting in the 40mer and 20mer are still unknown, it is tempting to speculate that such oligomerization behavior represents the ability of hPER2c in modulating the assembly of the large circadian protein complex.

In conclusion, we identified and characterized three different forms of recombinant hPER2c, 40mer, 20mer, and dimer. Our study provides experimental evidences to clarify the long-standing speculation about whether or not hPER2c indeed requires hCRYs to facilitate folding into a stable structure. Unlike the structure of mPER2c published before, our data clearly shows that hPER2c has very stable secondary structure and does not need to interact with other protein to fold. Despite the high homology between hPER2c and mPER2c, our results indicate significant differences in their thermal stabilities. The ability of hPER2c to form oligomers reflects its potentials in assembly of circadian protein complexes. The molecular mechanism of forming distinct oligomers with discrete sizes is unknown. High resolution structural determination of these forms of hPER2c is needed to fully understand the mechanisms of hPER2c oligomerization and its roles in the circadian rhythm.

## Supporting information

S1 Table Validation statistics of homology models of hPER2c from I-TASSER server. (DOCX)

S2 Table The secondary structure compositions of all three hPER2c forms estimated from their CD spectrum by three additional software. (DOCX)

S3 Table DLS data of hPER2c 40mer, 20mer, and dimer at temperature from 4°C to 85°C. (DOCX)

S1 Fig The effect of protein concentration on hPER2c stability and polymerization. (A) DLS profile of hPER2c 20mer measured as the protein was being concentrated. (B) DLS profile of hPER2c dimer measured as the protein was being concentrated.
(TIF)

S2 Fig The Z-average comparison of the hPER2c 40mer, 20mer, and dimer at various temperature. (TIF)

S3 Fig The effect of the salt on the stability and polymerization of hPER2c 20mer (A) and dimer (B).
(TIF)

S4 Fig The effect of the DTT and EDTA on the polymerization of hPER2c 40mer (A and B), 20mer(C and D) and dimer (E and F).
(TIF)

S5 Fig The alpha helix wheel plot of the longest helix in hPER2c model structure. Aliphatic residues are marked with blue squares; Hydrophilic residues are marked with red diamonds; Positively charged residues with black octagons. This wheel map is generated by EMBOSS pepwheel.
(TIF)
