# Supplementary material for: Thermal stability analyses of human PERIOD-2 C-terminal domain using dynamic light scattering and circular dichroism
Source: PLoS One. 2020 Apr 22;15(4):e0221180. doi: 10.1371/journal.pone.0221180 (PMC7176140; doi:10.1371/journal.pone.0221180)
Supplement: S1 Table — (DOCX) [file pone.0221180.s001.docx]

**S1 Table**. **Validation statistics of homology models of hPER2c from I-TASSER server.**

| Model | 1 | 2 | 3 | 4 | 5 |
| --- | --- | --- | --- | --- | --- |
| C-score^a^ | -2.33 | -2.74 | -2.36 | -2.86 | -3.00 |
| RMSD(Å) (Pruned atom pairs)^b^ | 0.60 (81) | 0.70 (82) | 0.66 (80) | 0.70 (81) | 0.65 (16) |
| RMSD(Å)^c^ | 0.83 | 0.76 | 0.96 | 0.83 | 15.37 |
| Helices percentage | 41.1% | 39.5% | 45.2% | 46.8% | 41.1% |
| Residues in most favored regions [A,B,L]^†^ | 74(67.3%) | 68(61.8%) | 71(64.6%) | 69(62.7%) | 70(63.6%) |
| Residues in additional allowed regions [a,b,l,p]^†^ | 27(24.5%) | 32(29.1%) | 31(28.2%) | 32(29.1%) | 31(28.2%) |
| Residues in generously allowed regions [~a,~b,~l,~p]^†^ | 2(1.8%) | 8(7.3%) | 4(3.6%) | 5(4.6%) | 7(6.4%) |
| Residues in disallowed regions^†^ | 7(6.4%) | 2(1.8%) | 4(3.6%) | 4(3.6%) | 2(1.8%) |
| Sum of residue in generously allowed and disallowed regions | 9 (8.2%) | 10(9.1%) | 8(7.2%) | 9(8.2%) | 9(8.2%) |

^a^ A confidence score for estimating the quality of predicted models provided by I-TASSER[1].

^b^ RMSD calculated by Chimera[2] match maker based on pruned atom pairs through iterations when hPER2c models were superimposed to mPer2 (PDB 4TC0).

^c^ RMSD calculated by Chimera match[2] command based on all 83 atom pairs between hPER2c models and mPer2 (PDB 4TC0).

^†^ Ramachandran plot statistics calculated by PROCHECK[3].
