# Supplementary material for: Thermal stability analyses of human PERIOD-2 C-terminal domain using dynamic light scattering and circular dichroism
Source: PLoS One. 2020 Apr 22;15(4):e0221180. doi: 10.1371/journal.pone.0221180 (PMC7176140; doi:10.1371/journal.pone.0221180)
Supplement: S2 Table — (DOCX) [file pone.0221180.s002.docx]

**S3 Table**. **The secondary structure compositions of all three hPER2c forms estimated from their CD spectrum by three additional software.**

| **Saftware** | **Dichroweb** | | | | | | |
| --- | --- | --- | --- | --- | --- | --- | --- |
|  | Helix1(%) | Helix2(%) | Strand1(%) | Strand2(%) | Turns(%) | Others(%) | NRMSD |
| **Dimer** | 3.53 | 6.47 | 16.60 | 9.60 | 20.33 | 40.23 | 0.09 |
| **20mer** | 2.93 | 3.73 | 32.27 | 12.80 | 13.13 | 33.07 | 0.30 |
| **40mer** | 7.67 | 8.47 | 13.93 | 10.70 | 14.80 | 25.73 | 0.14 |
| **Saftware** | **Jasco mSSE** | | | | | | |
|  | Hellx(%) | | Sheet(%) | | Turns (%) | Others(%) | NRMSD |
| **Dimer** | 7.8 | | 32 | | 14.6 | 45.6 | / |
| **20mer** | 11.6 | | 36.8 | | 16.5 | 35.1 | / |
| **40mer** | 17.5 | | 30.7 | | 15.9 | 35.9 | / |
| **Saftware** | **K2D3** | | | | | | |
|  | Hellx(%) | | Sheet(%) | | Turn(%) | Others(%) | NRMSD |
| **Dimer** | 51.69 | | 4.28 | | / | 95.72 | / |
| **20mer** | 66.85 | | 0.51 | | / | 99.49 | / |
| **40mer** | 76.84 | | 0.15 | | / | 99.85 | / |
