# Supplementary material for: Thermal stability analyses of human PERIOD-2 C-terminal domain using dynamic light scattering and circular dichroism
Source: PLoS One. 2020 Apr 22;15(4):e0221180. doi: 10.1371/journal.pone.0221180 (PMC7176140; doi:10.1371/journal.pone.0221180)
Supplement: S3 Table — (DOCX) [file pone.0221180.s003.docx]

**S5 Table**. **DLS data of hPER2c 40mer, 20mer, and dimer at temperature from 4°C to 85°C**

| Temperature(°C) | Dimer | | | 20mer | | | 40mer | | |
| --- | --- | --- | --- | --- | --- | --- | --- | --- | --- |
|  | Z-average  (d.nm) | PDI | Y-intercept | Z-average(d.nm) | PDI | Y-intercept | Z-average(d.nm) | PDI | Y-intercept |
| 4 | 13.74 | 0.57 | 0.79 | 19.67 | 0.24 | 0.91 | 28.56 | 0.26 | 0.92 |
| 10 | 11.35 | 0.42 | 0.77 | 21.87 | 0.25 | 0.92 | 31.23 | 0.25 | 0.92 |
| 15 | 11.19 | 0.40 | 0.79 | 21.32 | 0.26 | 0.91 | 30.89 | 0.25 | 0.92 |
| 20 | 14.56 | 0.53 | 0.79 | 21.36 | 0.25 | 0.91 | 31.00 | 0.25 | 0.92 |
| 25 | 13.12 | 0.47 | 0.79 | 21.35 | 0.27 | 0.91 | 30.50 | 0.24 | 0.92 |
| 30 | 11.39 | 0.37 | 0.73 | 21.90 | 0.30 | 0.91 | 30.77 | 0.25 | 0.92 |
| 35 | 12.62 | 0.39 | 0.74 | 21.55 | 0.27 | 0.91 | 31.18 | 0.24 | 0.92 |
| 40 | 13.84 | 0.41 | 0.76 | 22.27 | 0.26 | 0.91 | 32.09 | 0.24 | 0.92 |
| 45 | 20.56 | 0.30 | 0.75 | 23.10 | 0.25 | 0.91 | 34.24 | 0.25 | 0.91 |
| 50 | 21.26 | 0.38 | 0.79 | 26.87 | 0.31 | 0.91 | 37.25 | 0.24 | 0.91 |
| 55 | 28.01 | 0.49 | 0.81 | 30.64 | 0.27 | 0.91 | 42.07 | 0.20 | 0.90 |
| 60 | 30.64 | 0.39 | 0.84 | 35.60 | 0.26 | 0.91 | 52.32 | 0.18 | 0.93 |
| 65 | 44.38 | 0.29 | 0.88 | 48.51 | 0.23 | 0.92 | 64.22 | 0.17 | 0.92 |
| 70 | 64.15 | 0.22 | 0.89 | 63.21 | 0.19 | 0.94 | 80.35 | 0.13 | 0.90 |
| 75 | 97.06 | 0.17 | 0.91 | 86.81 | 0.15 | 0.92 | 108.07 | 0.12 | 0.93 |
| 80 | 184.77 | 0.08 | 0.91 | 125.13 | 0.14 | 0.94 | 154.87 | 0.14 | 0.92 |
| 85 | 877.63 | 0.35 | 0.91 | 198.23 | 0.16 | 0.93 | 288.33 | 0.28 | 0.92 |
