# Supplementary figures and images for: Thermal stability analyses of human PERIOD-2 C-terminal domain using dynamic light scattering and circular dichroism
Source: PLoS One. 2020 Apr 22;15(4):e0221180. doi: 10.1371/journal.pone.0221180 (PMC7176140; doi:10.1371/journal.pone.0221180)

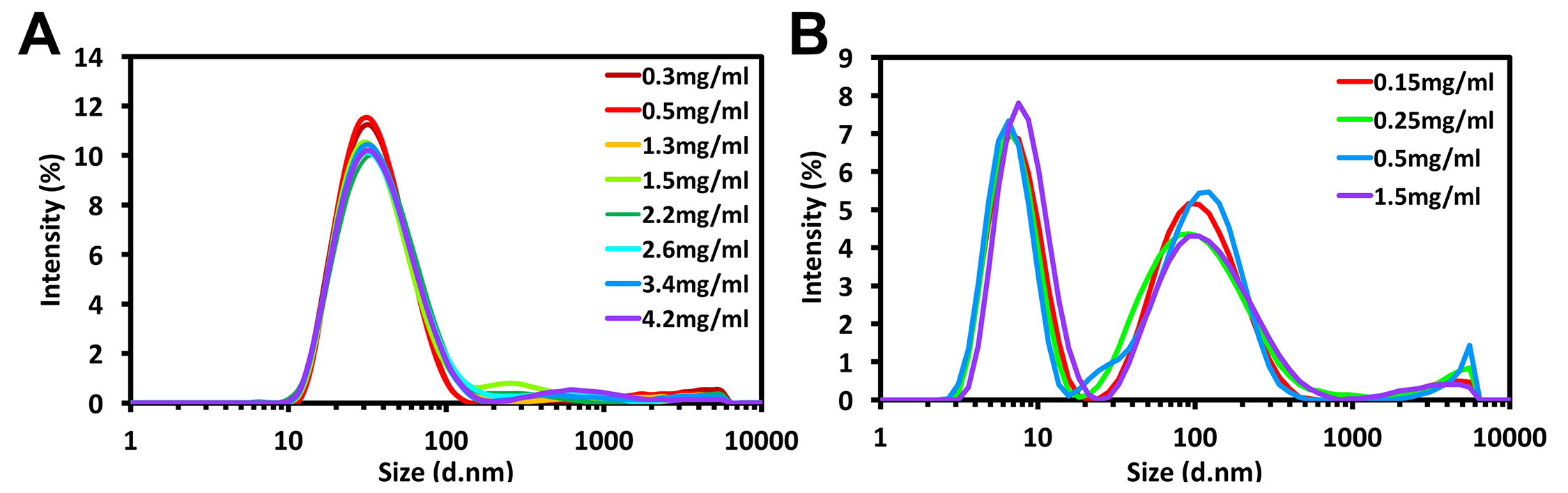

Supplement: S1 Fig — (A) DLS profile of hPER2c 20mer measured as the protein was being concentrated. (B) DLS profile of hPER2c dimer measured as the protein was being concentrated. (TIF) [file pone.0221180.s004.tif]

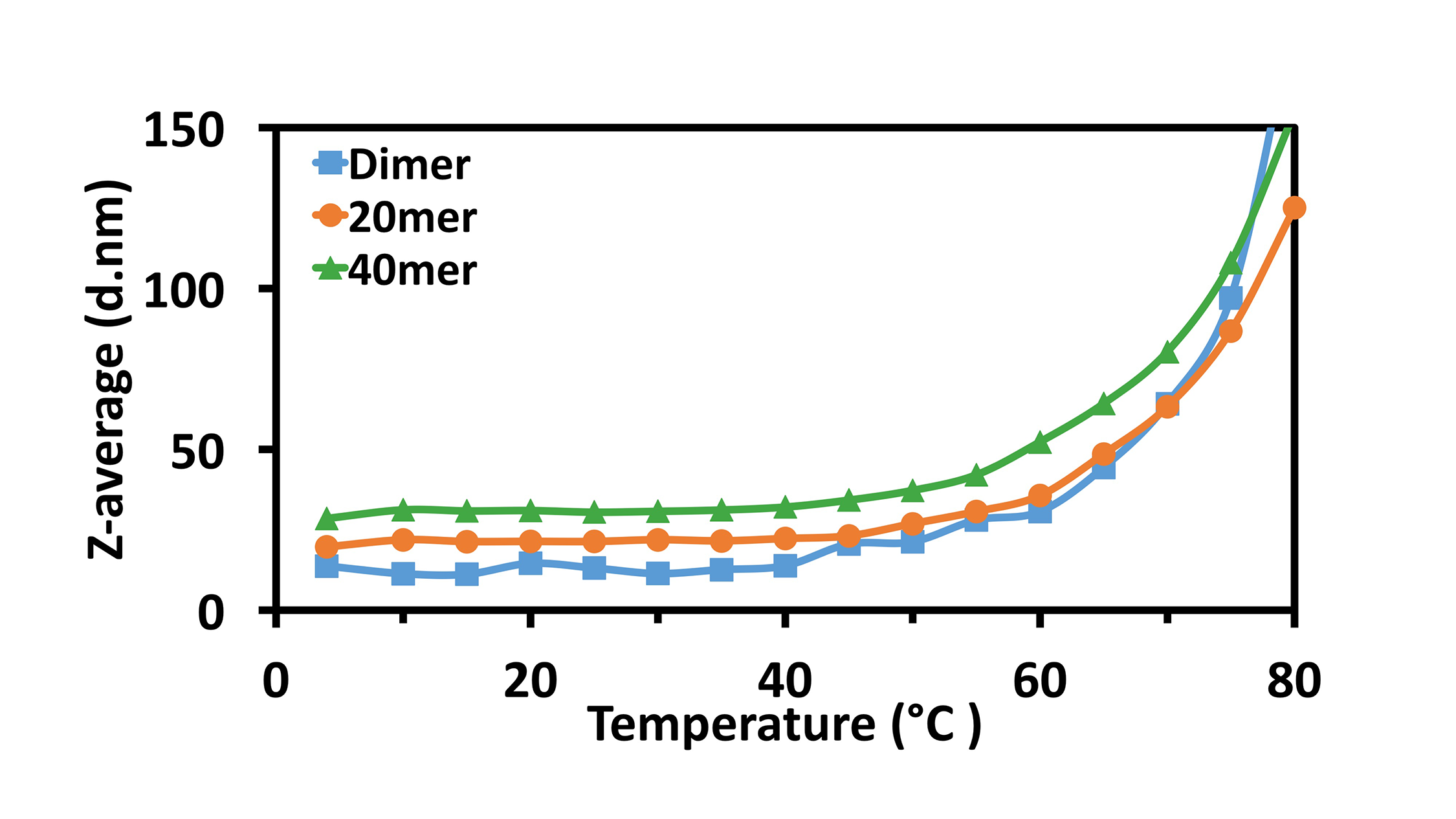

Supplement: S2 Fig — (TIF) [file pone.0221180.s005.tif]

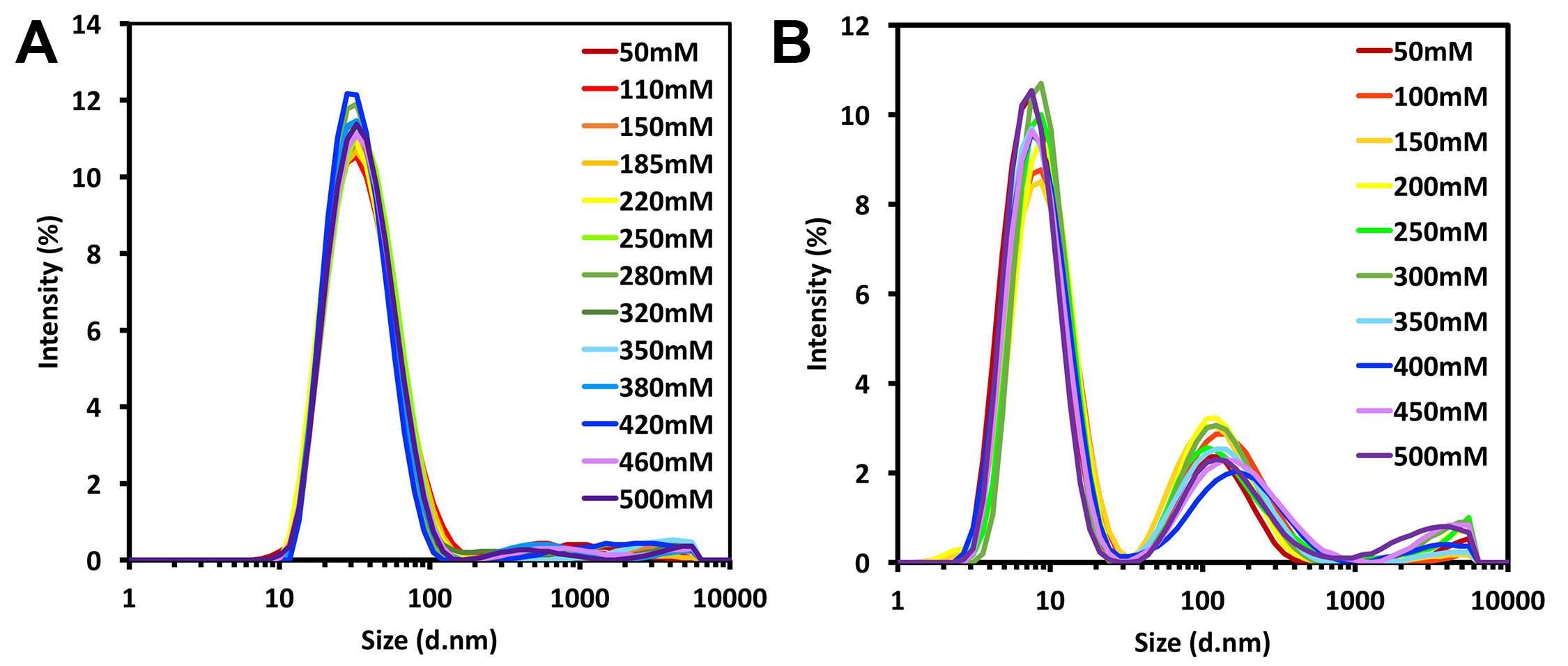

Supplement: S3 Fig — The effect of the salt on the stability and polymerization of hPER2c 20mer (A) and dimer (B). (TIF) [file pone.0221180.s006.tif]

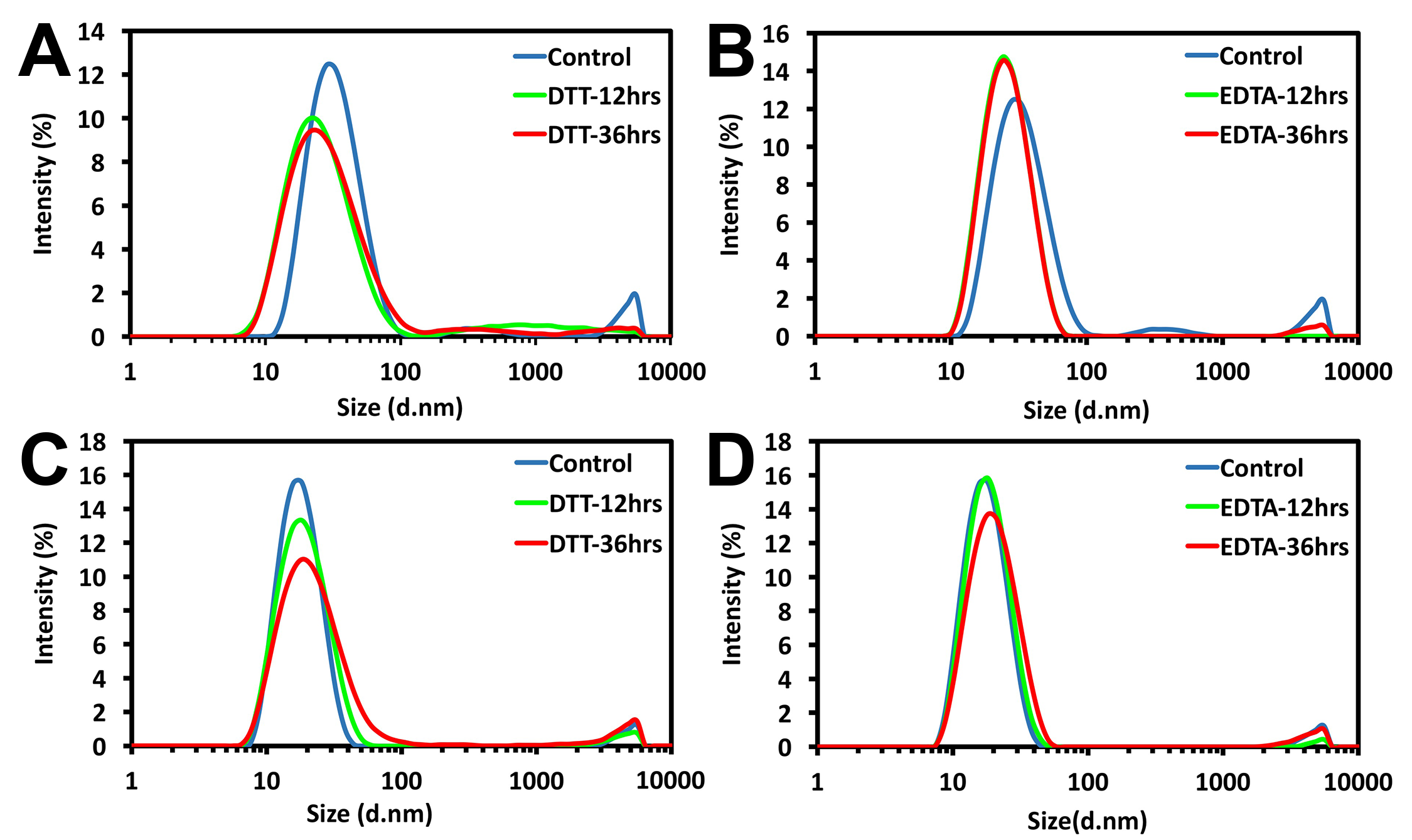

Supplement: S4 Fig — The effect of the DTT and EDTA on the polymerization of hPER2c 40mer (A and B), 20mer(C and D) and dimer (E and F). (TIF) [file pone.0221180.s007.tif]
